# Why do men who have sex with men practice condomless sex? A systematic review and meta-synthesis

**DOI:** 10.1186/s12879-022-07843-z

**Published:** 2022-11-14

**Authors:** Yan Shen, Ci Zhang, Maritta A. Valimaki, Hanzhu Qian, Leila Mohammadi, Yuanyuan Chi, Xianhong Li

**Affiliations:** 1 Xiangya School of Nursing, Central South University, Changsha, Hunan China; 2 Xiangya Center for Evidence-Based Nursing Practice & Healthcare Innovation, Changsha, Hunan China; 3 School of Public Health, Yale University, New Haven, CT USA; 4 College of Medicine and Public Health, Flinders University, Adelaide, SA Australia

**Keywords:** Men who have sex with men, Human immunodeficiency virus, Condom use, Barrier, Qualitative study, Meta-synthesis

## Abstract

**Background:**

Despite a large amount of behavioral interventions to reduce human immunodeficiency virus (HIV)-related high-risk sexual behaviors, consistent condom use remains suboptimal among men who have sex with men (MSM). However, current databases are lack of synthesized evidence to explain why MSM practiced condomless sex.

**Objective:**

Our study aims to conduct a systematic review and meta-synthesis of 39 eligible qualitative studies to explore the barriers to condom use among MSM.

**Methods:**

A systematic review and meta-synthesis of qualitative studies (1994–2021). On March 4, 2021, a comprehensive search was conducted in 14 electronic databases. The study was conducted based on the Joanna Briggs Institute’s recommendations.

**Results:**

Thematic analysis produced six synthesized themes, which were classified into three levels according to the Social-ecology Model. Individual level barriers to condom use included physical discomfort, lack of HIV/STI-related knowledge and substance use; interpersonal-level barrier was mainly the condom stigma, namely regarding using condom as symbols of distrust or HIV/sexually transmitted infections (STIs) prevention, or as violating traditional cognition of sex, or as an embarrassing topic; environmental/structural-level barriers included situational unavailability, unaffordability of condoms and power imbalance in the sexual relationship.

**Conclusion:**

This meta-synthesis offered in-depth understanding of condom use barriers for MSM and could guide the development of multifactorial interventions according to the identified barriers, especially targeting to reduce condom stigma, which has not been focused and intervened previously.

**Supplementary Information:**

The online version contains supplementary material available at 10.1186/s12879-022-07843-z.

## Introduction

In 2014, the Joint United Nations Programme on HIV/AIDS (UNAIDS) set a target to end acquired immune deficiency syndrome (AIDS) epidemic by 2030 [1]. However, despite the global efforts to prevent human immunodeficiency virus (HIV)/AIDS and treat people living with HIV/AIDS (PLWH), the HIV/AIDS epidemic continues to grow. Globally, the new HIV cases increased by 1.5 million in 2021 and 38.4 million people were living with HIV by the end of 2021 [2]. The growth of HIV/AIDS epidemic has been particularly alarming among men who have sex with men (MSM) [3]. The rate of new HIV infections among MSM has risen 25% worldwide between 2010 and 2019 [4]. In 2019, MSM were 26 times more likely to test positive for HIV compared to other adult men and accounted for 23% of new infections in the world [5].

It is well established that consistent and correct condom use was a highly effective preventive measure against HIV/AIDS and other sexually transmitted infections (STIs) transmission [6, 7]. However, despite a variety of behavioral intervention campaigns aimed to reduce unprotected sexual intercourse in MSM, consistent condom use remains suboptimal, and this was one of the reasons for HIV spread in this population [8]. Statistics from UNAIDS and meta-analyses showed that the rates of consistent condom use were 28% among HIV-infected and 29% among uninfected MSM in the United States [9], 61% among Canadian MSM, 64% among Australian MSM, 63% among Italian MSM, 39% among Egyptian MSM [10], and 47% among Chinese MSM [11]. In Sub-Saharan Africa and South/South-east Asia with greater burden of HIV among MSM, large-sample surveys also revealed that the rates of consistent condom use were even lower, 83.3% HIV-infected MSM in North Central Nigeria reported having condomless sex [12], 40.7% of MSM had condomless anal intercourse (CAI) in Bamako, Mali [13], 46.7% MSM reported recent non-condom sex in Bangkok, Chiang Mai and Phuket, Thailand [14], and 44.7% Vietnamese MSM reported not using a condom during their last anal sex [15].

Several systematic reviews summarized the effectiveness of the interventions on improving condom use, including motivational interviewing [16], mass media education [17], peer education [18], psychosocial support and counseling services [19–23], and condom social marketing [24]. Nevertheless, all these interventions showed short-term effects on reducing condomless sex, but no long-term effects [25–27]. Therefore, in order to develop effective interventions for increasing condom use among MSM, it is crucial to understand the barriers to condom use in a systematic perspective. Currently, both quantitative and qualitative studies have been conducted on this topic in different sociocultural settings [28–30], but comprehensive systematic reviews for synthesizing factors associated with condom use were still rare in MSM populations. For example, in MSM populations, we retrieved only one systematic review summarizing the structural barrier of price for condom use [31], and one meta-analysis showing that older age was associated a higher odd of condom use [32].

This study aims to aggregate, interpret, and synthesize the findings from a systematic review of the qualitative research literature about barriers to condom use among MSM. It is designed to address the question: Why do men who have sex with men practice condomless sex?

## Methods

### Conceptual framework

The Social-Ecological Model: A framework for Prevention (SEM) was chosen as the framework for conducting this systematic review. SEM was a useful framework to organize a comprehensive model of the factors influencing health related behaviors among key populations in order to thoughtfully inform effective interventions [33]. As studies have suggested that the barriers to condom use could be individual [34], interpersonal [35] or environmental factors [36], the model for guiding the meta-synthesis should combine these factors. The SEM is a proper model for the factors at individual/intrapersonal (e.g. psychology, knowledge, attitudes, behavior), interpersonal/network (social networks, social support) and environmental/structural (e.g. community, public policy, relationships among organizations/institutions, culture) levels that influence condom use among MSM [37].

### Study design

A systematic review was conducted to synthesize qualitative findings on the barriers to condom use among MSM. We focused on qualitative studies as the primary data source because individual perceptions were valuable resources for reasons not to use condom, and qualitative data could offer different perspectives to understand respondents’ perceptions on condom use using their own voices [38, 39].

The meta-synthesis was conducted according to the Joanna Briggs Institute (JBI) guidance for systematic reviews [40]. JBI is one of the world-famous evidence-based practice institutions. The Evidence-Based Practice (EBP) model they pioneered has been regarded as a benchmark indicator by the field of medical care. We used the JBI Qualitative Assessment and Review Instrument to synthesize evidence from individual qualitative studies to create a comprehensive understanding of the essence of the phenomenon [41]. The approach involved searching for articles meeting the inclusion criteria, assessing methodological quality, and synthesizing findings based on data extraction. The review protocol was registered in PROSPERO (CRD42020180894).

### Search strategy

We systematically searched PubMed, Web of Science, CINAHL, EMBASE, PsycINFO, Scopus, ProQuest, HMSS database, Elsevier/Science Direct, Cochrane, CNKI, Wanfang, VIP, and CBM for studies published in English and Chinese as of March 4, 2021. We used the search terms (“MSM”, “men who have sex with men”, “homosexual”, “gay”, “bisexual”, “same-sex”, OR “queer”) AND (“condom”, OR “condom use”) AND (“barrier”, “bias”, “obstacle”, OR “factor”) AND (“qualitative study”, “qualitative research”, “qualitative methods”, “interview”, “mix-methods study”, OR “mix-methods research”). Grey literature was also sought. Additional studies were hand searched by screening the references of included studies. The detailed literature search strategy used could be found in Table 1.

**Table 1:** Literature search strategy

*Databases searched*	
Pubmed, Web of Science (including MEDLINE), CINAHL, Embase, PsycINFO, Scopus, ProQuest, HMSS database, Elsevier/Science Direct, Cochrane, CNKI, Wanfang, VIP, CBM, NICE, OpenGrey, Google Scholar, AHRQ database, CDC database, Clinical Trials.gov, Abstracts of recent international conferences (e.g., International AIDS Society (IAS) et al	
*Setting*	
No restriction	
*Population*	
MSM OR men who have sex with men OR homosexual OR gay OR bisexual OR same-sex OR queer	
*Phenomena of interest*	
condom OR condom use	
*Methodology*	
qualitative study OR qualitative research OR qualitative methods OR interview OR mix-methods study OR mix-methods research	
*Search example in Pubmed*	
**#**	Searches
1	bisexuality/or heterosexuality/or homosexuality/or homosexuality, male/or transsexualism/
2	"Sexual and Gender Minorities"/
3	Sexually Transmitted Diseases/pc [Prevention & Control]
4	(bisexual* or homosexual* or "sexual dissent*" or gay* or queer* or "men who have sex with men" or MSM).tw,kf
5	or/1–4
6	Condoms/
7	(condom* or "safe sex" or "unsafe sex").tw,kf
8	6 or 7
9	interviews as topic/ or focus groups/ or narration/ or qualitative research/
10	((semi-structured or semistructured or unstructured or informal or "in-depth" or indepth or "face-to-face" or structured or guide? or group*) adj3 (discussion* or questionnaire*)).tw,kw
11	(Interview* or focus group* or diary or diaries or transcrib* or verbatim or field not* or memo? or memoing).tw,kw
12	((context* or semantic or content) adj2 analys*).tw,kw
13	(narrat* or qualitative* or ethnograph* or fieldwork or field work or field research* or informant* or phenomenolog* or hermeneutic* or grounded or interpretive* or participant observ* or mixed method* or background observ* or reflective* or reflection* or textual* or open-ended or theme? or thematic* or triangulat* or mixed method*).tw,kw
14	or/9–13
15	5 and 8 and 14
16	limit 15 to (Chinese or English)

FULL SEARCH uses bias OR inhibit* OR barrier* OR enabl* OR obstacle* OR facilitat* OR negotiat* OR prohibit* OR avoid* OR absence OR reduc* OR decreas* OR discomfort* OR uncomfort*

LIMITED SEARCH uses only bias OR inhibit* OR barrier* OR enabl* OR obstacle* OR facilitat* OR negotiat* OR prohibit* OR avoid* OR absence

We made updates to the registered protocol. The protocol title was changed from “*The barriers of condom use among men who have sex with men (MSM): A systematic review and meta-synthesis*” to “*Why do men who have sex with men practice condomless sex? A systematic review and meta-synthesis*”. Literature search was updated to 4 March 2021, so the number of included studies increased from 37 to 39.

### Eligibility criteria

Based on PIC(o)S terms, studies were included if all following criteria applied: (1) the target population (P) was men (at birth) having sex with men [14], with no age limit; (2) phenomenon of interest (I) was the barriers to condom use; (3) the study context (Co) was communities, associations, services, or public domains; and (4) the study design (S) employed a qualitative design or presented qualitative findings from a mixed method study.

Studies were excluded if they: (1) were statistical reviews, books or book chapters, letters, dissertations, editorials, or study protocols; (2) did not focus on condom use; or (3) only discussed the facilitators of condom use or effectiveness of interventions for improving condom use among MSM.

### Study selection

All included records were imported to Endnote X9 [YS, LM] and duplicates were identified [YS]. Two co-authors [YS, CZ] independently screened the titles and abstracts. Any disagreements were discussed by two reviewers or a third independent reviewer [XHL]. Full texts of the included abstracts were then read by two authors [YS, CZ]. Again, any disagreements were resolved through discussion.

### Quality assessment

JBI Critical Appraisal Skills Programme qualitative checklist [40] for qualitative studies was used to evaluate the quality of the included studies. The evaluation tool consists of 10 questions (Table 2). Each item of the tool was rated yes (Y), no (N), unclear (U), or not applicable (NA). Two reviewers [YS, CZ] independently assessed each study while the third reviewer [XHL] resolved any discrepancies. Consistent with prior reviews [42, 43], we set a priori inclusion criteria of at least six of the ten methodological quality indicators (Table 2).

**Table 2:** The quality evaluation of the included studies (*N* = 39)

Author and year	Q1	Q2	Q3	Q4	Q5	Q6	Q7	Q8	Q9	Q10	Quality (Y/10)
Li et al., 2010	Y	Y	Y	Y	Y	Y	N	Y	Y	Y	9/10
Moen et al., 2013	Y	Y	Y	Y	Y	Y	Y	Y	Y	Y	10/10
Peterson et al., 2003	U	Y	Y	Y	Y	Y	Y	Y	Y	Y	9/10
Taggart et al., 2017	Y	Y	Y	Y	Y	Y	N	Y	Y	Y	9/10
Campbell et al., 2013	Y	Y	Y	Y	Y	Y	Y	Y	U	Y	9/10
Mustanski et al., 2014	Y	Y	Y	Y	Y	Y	N	N	Y	Y	8/10
Ostergren et al., 2011	Y	Y	Y	Y	Y	Y	N	N	U	Y	7/10
Neville et al., 2016	Y	Y	Y	Y	Y	Y	N	Y	Y	Y	9/10
Schnarrs et al., 2012	Y	Y	Y	Y	Y	Y	Y	Y	U	Y	9/10
Tadele, 2010	Y	Y	Y	Y	Y	N	Y	Y	Y	Y	9/10
Li et al., 2016	Y	Y	Y	Y	Y	N	Y	Y	Y	Y	9/10
Beoughe et al., 2012	Y	Y	Y	Y	Y	N	N	Y	U	Y	7/10
Harawa et al., 2006	Y	Y	Y	Y	Y	Y	N	Y	U	Y	8/10
Valente et al., 2019	Y	Y	Y	Y	Y	Y	Y	Y	Y	Y	10/10
Chakrapani et al., 2013	Y	Y	Y	Y	Y	Y	Y	Y	U	Y	9/10
Adam et al., 2010	Y	Y	Y	Y	Y	Y	Y	Y	U	Y	9/10
Adam et al., 2000	Y	Y	Y	Y	Y	N	N	Y	U	Y	7/10
Adams et al., 2009	Y	Y	Y	Y	Y	Y	Y	Y	U	Y	9/10
Balán et al., 2009	Y	Y	Y	Y	Y	Y	N	Y	Y	Y	9/10
Boulton et al., 2010	Y	Y	Y	Y	Y	N	N	Y	U	Y	7/10
Diguez et al., 1996	Y	Y	Y	N	Y	Y	N	Y	U	Y	7/10
Eisenberg et al., 2011	Y	Y	Y	Y	Y	Y	Y	Y	Y	Y	10/10
Giano et al., 2019	Y	Y	Y	Y	Y	Y	Y	Y	Y	Y	10/10
Harawa et al., 2010	Y	Y	Y	Y	Y	Y	Y	Y	U	Y	9/10
Harper et al., 2016	Y	Y	Y	Y	Y	Y	N	Y	U	Y	8/10
Hospers et al., 1994	U	Y	Y	Y	Y	N	Y	Y	U	Y	7/10
Hubach et al., 2014	Y	Y	Y	Y	Y	Y	N	Y	U	Y	8/10
Klassen et al., 2019	Y	Y	Y	Y	Y	Y	Y	Y	U	Y	9/10
Kong, 2008	U	Y	Y	Y	Y	Y	N	Y	U	Y	7/10
Malebranche, 2009	Y	Y	Y	Y	Y	Y	Y	Y	Y	Y	10/10
Musinguzi et al., 2015	Y	Y	Y	Y	Y	Y	Y	Y	Y	Y	10/10
Middelthon, 2001	U	Y	Y	Y	Y	Y	Y	Y	U	Y	8/10
Siegler et al., 2014	Y	Y	Y	Y	Y	Y	N	Y	Y	Y	9/10
Starks et al., 2017	Y	Y	Y	Y	Y	Y	N	Y	U	Y	8/10
Zhang et al., 2018	U	Y	Y	Y	Y	N	N	Y	Y	Y	7/10
Wang et al., 2005	U	Y	Y	Y	Y	Y	N	Y	U	Y	7/10
Zhou, 2008	Y	Y	Y	Y	Y	Y	Y	Y	Y	Y	10/10
Ofreneo et al., 2020	Y	Y	Y	Y	Y	Y	Y	Y	U	Y	9/10
Rwstar et al., 2019	Y	Y	Y	Y	Y	Y	Y	Y	Y	Y	10/10

Y = Yes, N = No, U = Unclear, NA = Not applicable

Q1: Is there congruity between the stated philosophical perspective and the research methodology?

Q2: Is there congruity between the research methodology and the research question or objectives?

Q3: Is there congruity between the research methodology and the methods used to collect data?

Q4: Is there congruity between the research methodology and the representation and analysis of data?

Q5: Is there congruity between the research methodology and the interpretation of results?

Q6: Is there a statement locating the researcher culturally or theoretically?

Q7: Is the influence of the researcher on the research, and vice-versa, addressed?

Q8: Are participants, and their voices, adequately represented?

Q9: Is the research ethical according to current criteria or, for recent studies, is there evidence of ethical approval by an appropriate body?

Q10: Do the conclusions drawn in the research report flow from the analysis, or interpretation, of the data?

### Data extraction

Data were independently extracted from included studies by two reviewers [YS, CZ] and the results were compared and modified if needed. These data included: author, country, design, data collection, sample size and samples (Table 3). Data on the barriers to condom use were also extracted and are presented in Additional file 1.

**Table 3:** The characteristics of the included studies (*N* = 39)

Number	Author and year	Country	Study design	Data collection	*N*, Samples
1	Li et al., 2010	China	Ethnographic study	In-depth semi-structured interviews	17, MSM from diverse background
2	Moen et al., 2013	Tanzania	Ethnographic study	Participant observation and dialogical interviews	105, diverse same-sex-attracted men
3	Peterson et al., 2003	USA	Phenomenological research	Semi-structured interviews	75, African-American MSM
4	Taggart et al., 2017	USA	Phenomenological research	In-depth and semi-structured interviews	20, African-American MSM
5	Campbell et al., 2013	USA	Grounded theory approach	Qualitative interviews	48, same-sex male couples
6	Mustanski et al., 2014	USA	Mixed method research with Information-Motivation-Behavioral Skills model	Mixed method study, focus group interviews	75, adolescent gay and bisexual males
7	Ostergren et al., 2011	USA	Phenomenological research	Semi-structured interviews, thematic analysis	462, non-condom using MSM
8	Neville et al., 2016	New Zealand	Phenomenological research	Qualitative descriptive approach, Thematic analysis	960, MSM
9	Schnarrs et al., 2012	USA	Phenomenological research	In-depth, semi-structured interviews	75, men who engaged in bisexual behavior
10	Tadele, 2010	Ethiopia	Grounded theory approach	In-depth interviews and focus group discussion	24, MSM
11	Li et al., 2016	China	Descriptive and exploratory qualitative study design with the health belief model	In-depth semi-structured interviews, thematic analysis	17, MSM
12	Beoughe et al., 2012	USA	Grounded Theory approach	Semi-structured interviews	12, discordant gay couples
13	Harawa et al., 2006	USA	Phenomenological research	Semi-structured focus group interviews	30, African-American men have sex with men and women
14	Valente et al., 2019	Kenya	Phenomenological research	Semi-structured interviews	25, MSW (male sex workers) and 11 male clients of male sex workers
15	Chakrapani et al., 2013	India	Grounded theory research	In-depth interviews, focus-group discussions and key-informant interviews	93, MSM
16	Adam et al., 2010	Canada	Phenomenological research	In-depth semi-structured interviews	102, high-risk gay and bisexual men
17	Adam et al., 2000	Canada	Phenomenological research	Semi-structured one-on-one interviews	102, gay and bisexual men
18	Adams et al., 2009	New Zealand	Critical theory research	Face-to-face semi-structured individual interviews	22, MSM
19	Balán et al., 2009	USA	Phenomenological research	In-depth interviews	31, Latino MSM
20	Boulton et al., 2010	England	Sociological analysis	Open-ended interviews	78, gay men who engaged in anal intercourse without a condom
21	Diguez et al., 1996	USA	Phenomenological research with mixed method design	Semi-structured interviews	182, gays
22	Eisenberg et al., 2011	USA	Phenomenological research	Semi-structured in-depth interviews	34, young MSM
23	Giano et al., 2019	USA	Phenomenological research	Semi-structured interviews	40, MSM
24	Harawa et al., 2010	USA	Grounded theory with mixed method design	In-depth follow-up semi-structured interviews	17, MSM
25	Harper et al., 2016	USA	Phenomenological research	Semi-structured qualitative interviews	36, Black gay and bisexual young men living with HIV
26	Hospers et al., 1994	Holland	Phenomenological research	Focus group interviews	19, gay men who engaged in risk-taking behavior with casual partners
27	Hubach et al., 2014	USA	Grounded theory approach	Semi-structured interviews	77, behaviorally bisexual men
28	Klassen et al., 2019	Canada	Social ecological analysis	Semi-structured interviews	19, gay men
29	Kong, 2008	China	Phenomenological research	In-depth semi-structured face-to-face interviews	30, MSW
30	Malebranche, 2009	USA	Phenomenological research	Semi-structured, one-on-one interviews	29, self-identified Black MSM
31	Musinguzi et al., 2015	Uganda	Phenomenological research	Semi-structured interviews	85, self-identified adult MSM
32	Middelthon, 2001	Norway	Phenomenological research	Repeated in-depth interviews	20, young gay men
33	Siegler et al., 2014	South Africa	Phenomenological research	In-depth interviews	34, South -African MSM
34	Starks et al., 2017	USA	Phenomenological research with thematic analysis	Semi-structured interviews	17, HIV-negative gay male couples
35	Zhang et al., 2018	China	Phenomenological research	Semi-structured interviews	35, male students who have sex with men
36	Wang et al., 2005	China	Phenomenological research	Focus group discussion, individual interview, observation	Unclear, MSM from five cities
37	Zou, 2008	China	Phenomenological research with mixed method design	Individual in-depth interviews	20, MSM
38	Ofreneo et al., 2020	Philippines	Critical realist inquiry	Semi-structured interviews	17, MSM
39	Rwstar et al., 2019	Philippines	Situated socio-ecological perspective research	Semi-structured interviews	23 transgender women and 7 cisgender MSM

### Data synthesis

The thematic synthesis approach outlined by Thomas and Harden [44] was broadly followed to identify, interpret, and explain the findings of the original studies [45]. This approach consists of three steps: coding the original descriptions line by line, developing descriptive themes, and generating analytical themes [44]. In our review, the steps were as follows:

First, to determine the barriers to condom use among MSM, findings from each original qualitative study were extracted and coded using the JBI-Qualitative Assessment and Review Instrument [41]. Original findings from the included studies were repeatedly re-examined, compared, and discussed by the study team to obtain final codes. Second, based on a thorough understanding of these codes, similar codes were combined to generate new categories called “descriptive themes” [44]. Third, descriptive themes were further categorized based on similarity or differences in meanings and subjected to meta-synthesis to produce aggregated findings called “analytical themes” [41]. All “analytical themes” were supported by the raw data quotes [46]. Each step was independently completed and cross-checked by two reviewers [YS, CZ]. Any disagreements were discussed and solved by the team.

## Results

### Study selection and characteristics

**Fig. 1:**
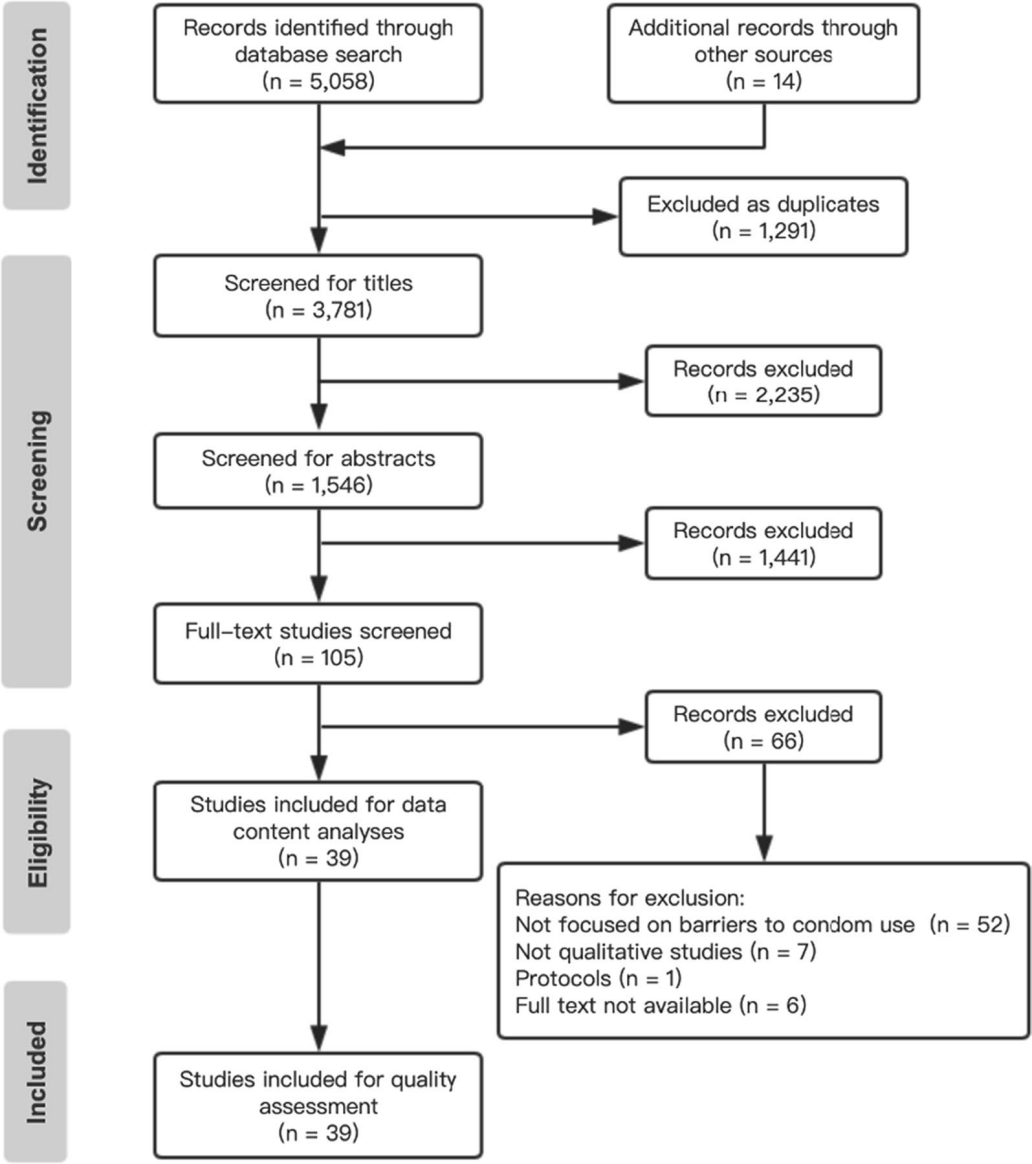
Flow chart of study selection

The electronic literature search identified 5072 records. After the removal of 1291 duplicates, 3781 records remained. Subsequently, 3676 records were removed after reviewing titles and abstracts, as they did not contain data on barriers to condom use among MSM. We further screened all 105 full-text articles and identified 39 articles for inclusion in the synthesis (Fig. 1).

The included studies (N = 39) were conducted between 1994 and 2021. Most studies (82%) were conducted in upper-middle and high-income countries. Thirty-five studies (90%) employed a qualitative design while four (10%) used a mixed method approach. Data were typically collected using in-depth semi-structured interviews (82%), and 10% of the studies used focus group discussion. Sample size varied from 12 to 960. Detailed characteristics of the studies are described in Table 3.

### Quality of the studies

All 39 studies met the cutoff of the quality assessment, which was mentioned in the “Methods” section and were thus included in the review (Table 2).

### Data synthesis

**Fig. 2:**
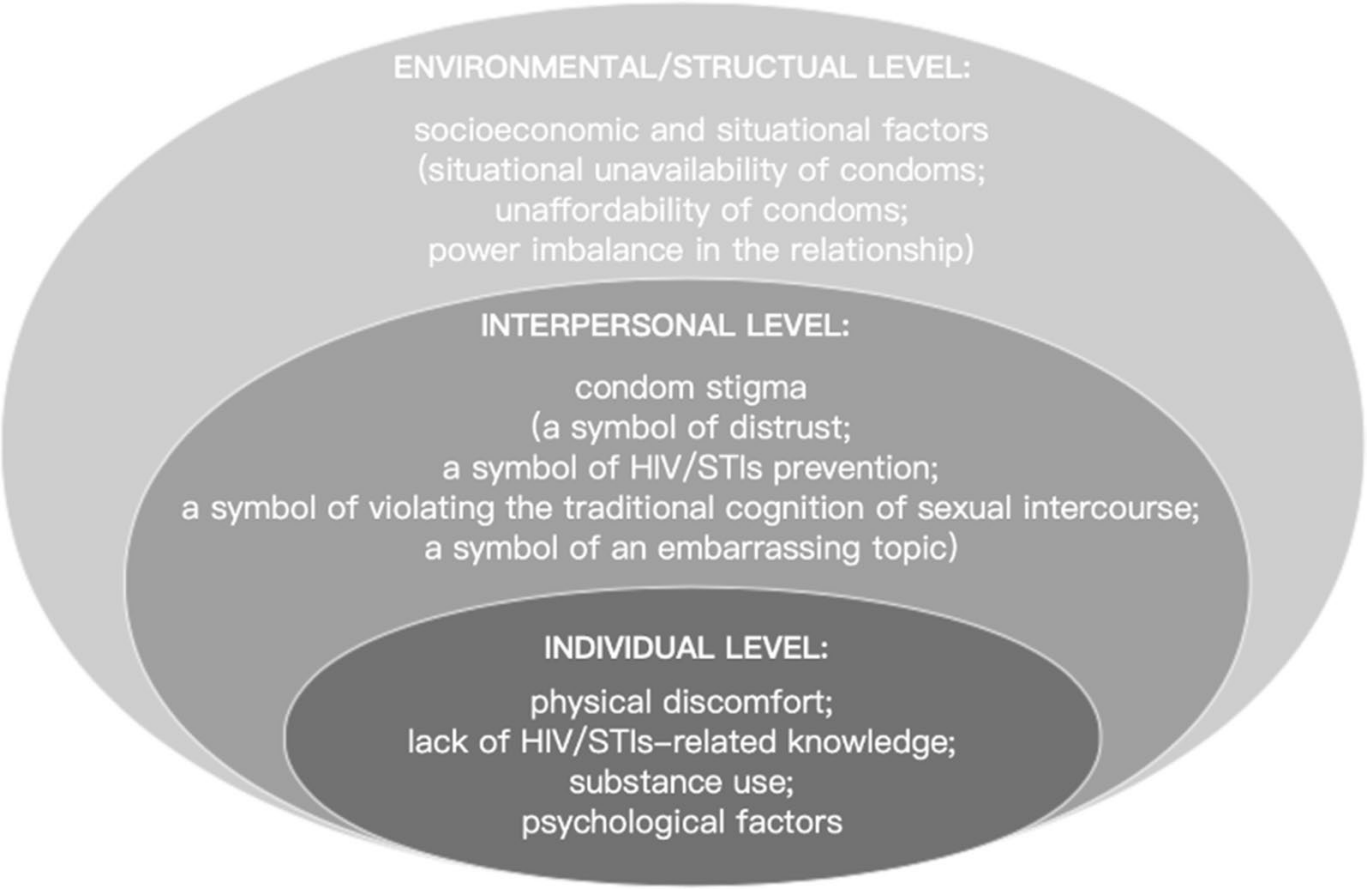
Barriers to condom use in three levels according to the Social-ecology Model

A total of 423 original findings relevant to condom use barriers were extracted (Additional file 1). Thematic analysis of the original findings produced six synthesized themes, which were classified into three levels according to the Social-ecology Model. Physical discomfort, lack of HIV/STI-related knowledge, substance use and psychological factors were the individual-level barriers; condom stigma, including regarding using condom as symbols of distrust, HIV/STIs prevention, violating traditional cognition of sex, and embarrassing topic were interpersonal-level barriers; socioeconomic and situational factors, including situational unavailability, unaffordability of condoms, and power imbalance in the relationship were environmental/structural-level barriers (Fig. 2).

### Domain 1: individual-level barriers

#### Physical discomfort

Thirty-two studies indicated that physical discomfort diminished consistent condom use (Table 4). Specifically, physical pain was a very common reason for not using condoms.

“Condoms are not bad, but the problem is when used for more than a minute, they tend to get dry; it starts hurting and can even cause bruises. It is good to use a condom for a few minutes and then get a new one.” (Musinguzi et al., 2015, P. 5) [28]

**Table 4:** Contribution of individual review items to final synthesized themes and sub-themes (N = 39)

Number	1	2	3	4	5	6	7	8	9	10	11	12	13	14	15	16	17	18	19	20
	Li et al. 2010	Moen et al., 2013	Peterson et al., 2003	Taggart et al., 2017	Campbell et al., 2013	Mustanski et al., 2014	Ostergren et al., 2011	Neville et al., 2016	Schnarrs et al., 2012	Tadele, 2010	Li et al., 2016	Beoughe et al., 2012	Harawa et al., 2006	Valente et al., 2019	Chakrapani et al., 2013	Adam et al., 2010	Adam et al., 2000	Adams et al., 2009	Balán et al., 2009	Boulton et al., 2010
*Individual-level*																				
**Physical discomfort**																				
Physical pain		×				×	×		×		×		×					×	×	
Reduced sexual pleasure	×	×	×			×	×	×	×	×		×	×	×	×	×			×	×
**Lack of HIV/STI-related knowledge**		×	×			×		×	×	×	×	×	×		×	×	×	×		×
**Substance use**			×				×		×	×			×	×		×	×		×	×
**Psychological factors**																				
Fluke thinking										×		×		×	×					×
Negative emotions	×										×					×	×			×
Vengeful perspective															×				×	
*Interpersonal-level*																				
**Condom stigma**																				
A symbol of distrust	×		×	×		×	×	×			×	×			×	×	×	×		×
A symbol of HIV/STIs prevention	×	×	×		×	×	×	×		×	×	×			×	×	×	×	×	×
A symbol of violating the traditional cognition of sex	×	×	×	×			×								×	×		×	×	
Feeling embarrassed to initiate condom related communication		×	×		×	×				×					×				×	×
*Environmental/structural-level*																				
**Socioeconomic factors**																				
Situational unavailability			×		×	×	×		×		×			×	×	×	×	×		×
Unaffordability of condoms		×	×			×	×				×		×	×					×	
Power imbalance in the relationship											×		×	×	×					×

Number	21	22	23	24	25	26	27	28	29	30	31	32	33	34	35	36	37	38	39
	Diguez et al., 1996	Eisenberg et al., 2011	Giano et al., 2019	Harawa et al., 2010	Harper et al., 2016	Hospers et al., 1994	Hubach et al., 2014	Klassen et al., 2019	Kong, 2008	Malebranche, 2009	Musinguzi et al., 2015	Middelthon, 2001	Siegler et al., 2014	Starks et al., 2017	Zhang et al., 2018	Wang et al., 2005	Zou, 2008	Ofreneo et al., 2020	Rwstar et al., 2019
*Individual-level*																			
**Physical discomfort**																			
Physical pain			×								×					×	×		
Reduced sexual pleasure	×	×	×	×	×	×	×	×			×	×	×	×	×	×			
**Lack of HIV/STI-related knowledge**			×		×		×	×	×	×	×				×	×	×		×
**Substance use**	×		×			×	×	×			×		×		×		×		
**Psychological factors**																			
Fluke thinking	×							×									×		
Negative emotions						×				×									
Vengeful perspective				×															
*Interpersonal-level*																			
**Condom stigma**																			
A symbol of distrust	×	×	×	×		×	×	×	×	×	×	×	×	×	×	×	×	×	
A symbol of HIV/STIs prevention		×	×	×		×		×		×	×			×	×	×	×	×	×
A symbol of violating the traditional cognition of sex								×						×					×
Feeling embarrassed to initiate condom related communication	×	×							×		×						×		×
*Environmental/structural-level*																			
**Socioeconomic factors**																			
Situational unavailability	×	×		×	×		×	×		×	×		×	×		×	×		×
Unaffordability of condoms				×		×			×		×					×	×		×
Power imbalance in the relationship			×					×							×				

Reducing sexual pleasure was another common complain reported in 29 studies (Additional file 1). MSM complained that condoms reduced physical sensation and diminished sexual pleasure. In order to avoid reducing sexual pleasure, delaying ejaculation, and diminishing their capabilities or sensitivity, MSM preferred not to use condoms during intercourse.

“My opinion is that men do not like to use condoms because they take away the pleasure of the actual flesh.” (Harawa et al., 2006, P. 5) [34]

#### Lack of HIV/STI-related knowledge

The findings from 25 studies (Table 4) suggested that some MSM were unclear about the necessity of condom use to prevent HIV and other STIs. Some MSM knew very little about the exact prevalence of HIV among MSM and believed it could not happen to them. Owing to a gap in sexual education and incorrect knowledge of HIV/STIs and condoms, nearly one-third of MSM were suspicious about condoms. Nine studies illustrated that as a result of inaccurate knowledge, MSM had developed their own ways to prevent HIV infection.

“So, I asked him why he agreed not to use a condom, and he told me that he just went to the toilet and took the sperms out afterwards. Actually, I also used to think that sex between men is safer since you can remove the sperms afterwards. I used to believe that until a friend told me that this is not the case.” (Moen et al., 2013, P. 11) [47]

#### Substance use

Nineteen studies reported substance use as a barrier to condom use (Table 4). Intoxication and the effect of drugs including rush poppers, methamphetamine and heroin made respondents lose their self-control and decision-making capacity with regard to condom use.

“I think that...the reason most men don’t use condoms is that they are either intoxicated or on some type of drug. Caught up in the heat of the moment, they lose self-control and don’t stop to think (whether they should use condoms or not).” (Harawa et al., 2006, P. 6) [34]

#### Psychological factors

Sixteen studies showed that psychological factors, including “fluke thinking”, negative emotions, and a vengeful perspective, contributed to condom-less sex (Table 4).

Fluke thinking refers to the psychological activities of accidentally obtaining benefit and success, avoiding misfortune, or exemption from disaster [48]. For example, some MSM were aware of high risk of HIV/STIs, but they believed that it could not happen to them. Eight studies demonstrated that “fluke thinking” was a significant psychological factor negatively affecting condom use (Table 4).

“Although I have heard about the seriousness of HIV, I never thought I would be unlucky enough to be infected. Although I was worried, there was still a fluke mind for myself. I thought I could get away with it.” (Zou, 2008, P. 38) [49]

In seven studies, participants stated that their negative emotions were an important factor in risk-taking behavior. Bad moods, negative emotions, and daily pressure were regarded as barriers to safe sex, mainly owing to low self-esteem because of their sexual minority identities (Table 4).

“When my self-esteem is down...or if I’m depressed and just sort of, you know, feeling downtrodden by the world…it’s just, I...get into that ‘I don’t care’ mode (even without condoms).” (Adam et al., 2010, P. 5) [50]

Although not common, three studies demonstrated that MSM decided not to use condoms from a vengeful perspective, because they had been unexpectedly infected with HIV (Table 4).

“A person could feel, ‘Someone didn’t tell me they had a disease, so I caught it from them. So now, I’m going to give it to everybody I can.’ You know?” (Harawa et al., 2010, P. 13) [51]

### Domain 2: interpersonal-level barriers

Nearly all included studies (n = 35, Table 4) implied that condom stigma had a negative influence on condom use among MSM. Condom stigma refers to any taboos or misbeliefs about condom use or feeling ashamed or embarrassed to talk about using condoms. This was demonstrated through four sub-themes.

#### A symbol of distrust

Thirty studies indicated that concerns regarding trust and loyalty were the primary reason for non-use of condoms (Table 4). Unprotected anal intercourse was usually interpreted as a primary sign of trust and intimacy. Proposing condom use during intercourse aroused suspicions about disloyalty.

“It is based on respect, affirmation, and trust for your partner. Let’s suppose you want to be his boyfriend, and if you used a condom or required him to use one, it sends the message that you do not trust him. It is like an insult.” (Li et al., 2016, P. 7) [35]

Especially, having a regular sexual partner or being in a monogamous relationship were reasons not to use condoms. Participants viewed sexual monogamy as a buffer against the risk of HIV/STIs acquisition within the relationship, and condom use was seen as an indicator of an inferior relationship.

“Why didn’t I wear a condom? Because I was either in a committed relationship with that person or had known that person long enough not to question him when he told me about his sexual past.” (Mustanski et al., 2014, P. 6) [52]

#### A symbol of HIV/STIs prevention

Twenty-nine studies indicated that MSM usually felt that condoms are solely for HIV/STIs prevention (Table 4). In other words, once MSM believed their partners were “safe” (without HIV infection), they no longer used condoms. On the contrary, initiating condom use automatically brought thoughts of HIV-related risk to the fore. Therefore, condoms served as a reminder of the possibility of HIV/STIs.

“It’s expected, routine, not to use a condom, because if we did, it would imply that one of us was infected or had sex outside the relationship.” (Boulton et al., 2010, P. 7-8) [53]

Nine studies further showed that MSM might use some techniques to assess their partners’ health to avoid the embarrassment of talking about HIV or using condoms. These techniques included observing their partner’s physical conditions (such as physical appearance), assessing their partner’s living situation, and checking their partner’s sexual history. They could also adopt the strategy of “sero-positioning” or “serosorting” (according to the HIV serostatus and/or sex role) [54] to decide whether to use condoms.

“I went to his home. It was a big apartment. We didn’t use condoms because I felt that he would not be an unsanitary person, and his body condition was healthy.” (Li et al., 2010, P. 5) [55]

Treatment optimism contributed to HIV-related high-risk behaviors as well. Given the availability of highly effective antiretroviral treatment, HIV has come to be regarded as a treatable chronic disease. Some MSM no longer had a fear of HIV and therefore might expose themselves to the risk of infection in condomless sex.

“Most people are aware of the risk factors for HIV, including not using condoms. I know people who think that HIV medication will fix things. There are a lot of gay men who think that HIV is curable, and because of that [they] take risks and don’t use condoms.” (Neville et al., 2016, P. 14) [56]

#### A symbol of violating the traditional cognition of sexual intercourse

Twelve studies reported that MSM usually hold the traditional cognition of sexual intercourse and believe that using condoms is a violation of its true purpose (Table 4). In some settings, they believed that sexual intercourse is a symbol of “true love” and must involve direct genital contact; this is known as “*rouyu*” (*desire of the flesh*) or “bare sex.” There is a belief that during intercourse, partners should exchange body fluids. Based on this traditional cognition, condom use was deemed as violating the true meaning of human intercourse.

“At its root, love is direct flesh-to-flesh contact; that’s so-called *‘rouyu.’*” Two lovers should blend in with each other.” (Li et al., 2010, P. 3) [55]

#### A symbol of an embarrassing topic

Fourteen studies showed that MSM felt embarrassed to suggest using a condom or even to initiate the discussion regarding condom use (Table 4). In some situations, although they tried to initiate a condom-related discussion, miscommunication led to awkwardness. Furthermore, buying condoms was a huge challenge, especially for young MSM. They felt ashamed to go to the store to buy condoms and did not feel smart enough as they could not determine the kind of condoms to get. They complained that cashiers gave them dirty looks because of their young appearance. Some unmarried men said they felt embarrassed to carry condoms and feared discovery by their parents or others.

“For example, I would be extremely embarrassed to ask for them (condoms), and wouldn’t even know where to get them (I think they’re sold in vending machines and pharmacies). Also, some [people] don’t know how to use them properly and would feel awkward to use them.” (Mustanski et al., 2014, P. 6) [52]

### Domain 3: environmental/structural-level barriers

Thirty-one studies revealed that socioeconomic and situational factors were an insurmountable obstacle to consistent condom use (Table 4). Socioeconomic and situational factors were spread across three sub-themes: situational unavailability of condoms, unaffordability of condoms, and power imbalance in the relationship.

### Situational unavailability of condoms

Evidence of situational unavailability was identified in 25 studies (Table 4). In five studies, participants experienced unplanned sex with no condom at hand. Furthermore, the “heat of the moment,” “not enough condoms,” and “unavailability of appropriately sized condoms” also contributed to the low rate of consistent condom use.

“I don’t carry condoms with me but if the other person has them, I don’t resist using them. But I know that others also don’t carry condoms with them so then most of the time we have sex without condoms.” (Chakrapani et al., 2013, P. 7) [57]

#### Unaffordability of condoms

Fifteen studies reported that despite being aware of the benefits, some MSM, particularly those who were homeless, could not afford condoms, whether of the regular type or of particularly good quality (Table 4). In some studies, MSM could get free condoms, but most of them complained that these were of poor quality, and some even experienced condom breakage or slippage and other quality deficits.

“I never used condoms because I didn’t have money to buy them or lacked both money and place to acquire them.” (Musinguzi et al., 2015, P. 5) [28]

#### Power imbalance in the relationship

In eight studies, there were imbalances in participants’ relationship power dynamics and sexual decision-making (Table 4). Some explained that they lacked the ability to put their point across, while others experienced sexual abuse and were forced to have unprotected intercourse. Moreover, male sexual workers who served male clients would engage in unprotected sex to earn more money.

“I don’t want it (not to use condoms), but if he gives more money, I think it’s OK.” (Kong, 2008, P. 3) [36]

## Discussion

This review and meta-synthesis had qualitatively presented the comprehensive barriers to condom use among MSM in global community settings. However, no geographical differences in reported barriers were found in this review. Based on the analyses of qualitative data extracted from included studies, our results provide insight into the barriers that influenced MSM’s use of condoms at individual, interpersonal, and environmental/structural levels. Multidimensional understanding of the condom using barriers could provide strategies for researchers, health providers and policy makers to reduce high-risk sexual behaviors among MSM and contribute to achieving the 2030 target of ending HIV epidemic.

Individual-level barriers were commonly reported in literature. In these synthesized results, the most common complain on why taking condomless sexual behaviors was that the usage of condom during sex made them felt pain, uncomfortable and reduced sexual pleasure. However, the description of physical discomfort and sexual pleasure is subjective and cannot be objectively measured by tools, thus some scholars believed it might be susceptible to psychological influence [58]. Pachankis et al. [59] revealed that psychological stress, especially sexual minority stress, had a direct and considerable impact on their HIV-related risk behaviors among MSM. Some MSM viewed enjoyment of sexual pleasure as a way of escaping from sexual minority stress [60–63]. Zou [49] also noted that MSM might prioritize sexual pleasure over sexual safety. Therefore, addressing psychological stress through other measures could potentially reduce the chance of relying on enjoining sexual pleasure to achieve temperately joy among MSM.

Not surprisingly, lack of HIV/STI-related knowledge was identified as an individual-level barrier to condom use, especially in resource-limited countries and areas with high stigma towards HIV and homosexuality [64]. However, the gap between knowledge and practice still exists, and better knowledge does not always lead to safer sexual practices [65]. Literature indicated that the reasons might be rooted in culture, values, individual feelings, and other social-economic-psychological factors [65–68]. Another study [35] also showed that sub-cultural factors had a huge impact on misbelieves about HIV transmission, which greatly affected safe sex in MSM. Therefore, intervention programs for improving the HIV related knowledge should fill the knowledge-behavior gap by taking into account of the social-economic and subcultural characteristics.

In addition, condom stigma was synthesized as a prominent barrier to condom use in interpersonal level. “Stigma” is originally a Greek term referring to “bodily signs designed to expose something unusual and bad about the moral status of the signifier” [69], like a tattoo or a mark on a slave. Goffman further defined stigma as “a characteristic or an attribute that is deeply discrediting” [69]. As per the evidence synthesized in this review, condom stigma was a perceived negative attitude and characteristic about condom use by MSM. Several studies have demonstrated that attitude toward condoms was an important variable in predicting condom use behaviors [70]. In our analysis, we found that condom stigma was mainly derived from the distortion of trust and loyalty relationship, viewing condom as a symbol as HIV/AIDS and STIs, shame and embarrassment of sex-related topic, and distortion of cognition of sexual intercourse, which were classified into four sub-themes. Condom use and even discussions about safe sex were regarded as a symbol of disloyalty or distrust between partners. Some people avoid using condoms because of the belief that it violates the true purpose of human intercourse. Owing to the desire to be accepted in one’s social network, the fear of being stigmatized may be a stronger driver of condomless sex than the commitment to safe sex in this population.

Furthermore, socioeconomic and situational factors that were classified at environmental/structural level barriers to condom use [28, 71–73]. Socioeconomic vulnerability leads to less power to negotiate safer sex for some MSM, for example the money boys (or male sexual workers) and some young men who were unemployed [73]. Moreover, situational sex is common in the MSM community, while men might not have condom by hands, which also results in unprotected sex [35]. Even worse when group substance using happened in some circumstances, which greatly increases the likelihood of unprotected intercourse [74–76].

There were also some other factors not outlined in our qualitative synthesis. For instance, studies revealed that MSM who were willing to use PrEP reported that they would not use condoms while taking PrEP [77, 78], and MSM who used post-exposure prophylaxis (PEP) were also less likelihood to use condoms [79]. Although PrEP and PEP were effective biomedical HIV prevention approaches, it was recommended that they should be used in combination with other HIV prevention methods such as condoms and testing and treatment of other STIs [80]. Treatment as prevention (TasP) strategy is also recommended for people living with HIV (PLWH), as PLWH who take anti-HIV medication as prescribed and maintain an undetectable viral load cannot transmit the virus sexually, which is called as undetectable equals untransmittable (U = U) [81, 82]. However, willingness to use and accessibility of these strategies were suboptimal. For example, the willingness to use PrEP by MSM was 58.6% worldwide [83], and the actual PrEP uptake rate was just 28% in low- and middle-income countries [84]. In addition, these strategies could not prevent other STIs. Therefore, combining condom use with these approaches is likely to enhance the efficacy of HIV prevention and reduce the risk of other STIs [14]. Besides, marital status and relationships were also reported as influencing factors toward condom use [85]. Among bisexual men who had female sexual partners, they might not use condoms if a female sexual partner was on birth control or could not become pregnant [86]. MSM also tended to have unprotected sex with their regular sexual praters, because unprotected sex could be a symbol of the trust in the relationship, which was summed up in condom stigma in our study. In addition, male sex workers (MSW) often engaged in condomless sex with their commercial partners under the request of their client due to the imbalanced power of the relationship [87], which was also synthesized in the relationship’s power imbalance in our study.

This review had several limitations. First, despite the comprehensive search strategy, some qualitative studies might have been excluded because of the language restrictions to Chinese and English in our analysis; this may lead to selection bias. Second, the synthesized themes generated from the qualitative results were based on our subjective discussions which might be limited by personal comprehension. However, triangulation was adopted to ensure the credibility of the results, and the data were categorized by two persons and checked by a third person in the study group. Third, all the included articles in this systematic review did not distinguish MSM with transgender women (TGW), thus future original studies could explore the unique barriers and facilitators on condom use for TGW. Finally, our review focused only on barriers of condom use during data extraction process, and did not included study findings on the facilitators for condom use, which is the other side of the study topic. It might be worthwhile to conduct a separate research on it.

Our review and meta-synthesis presented the comprehensive barriers to condom use among MSM and identified that barriers were deeply influenced by individual, intrapersonal and social-structural level factors. Our results could offer deeper insight into what kind of factors should been taken into account when designing innovative and long-term effective interventions to improve safer sex practices among MSM [88]. Future interventions could target on a specific barrier or collectively focusing on several barriers, for example, condom stigma has been synthesized as a new concept in this meta-synthesis, however, rare interventions has been conducted to address condom stigma. Thus, future studies can focus on how to reduce condom stigma among MSM from sub-cultural perspective to improve safer sex.

## Conclusion

This is the meta-synthesis to qualitatively summarize the barriers to condom use among MSM. The social-ecological model provides a relevant framework to understand and analyze the barriers that affect condom use among MSM, which can be classified into six themes at three levels. Based on the findings, scholars and health policymakers can develop tailored, innovative and effective interventions to address condom use barriers and reduce HIV transmission risk among MSM globally.

## Supplementary Information

**Additional file 1.** Original findings of the included studies (N = 39).

## Data Availability

All data generated or analyzed during this study are included in this published article and its additional information files.

## Abbreviations

MSM: Men who have sex with men
HIV: Human immunodeficiency virus
AIDS: Acquired immune deficiency syndrome
PLWH: People living with HIV/AIDS
STIs: Sexually transmitted infections
UNAIDS: The Joint United Nations Programme on HIV/AIDS
SEM: The social-ecological model
JBI: Joanna Briggs Institute
EBP: The evidence-based practice
MSW: Male sex workers
PrEP: Pre-exposure prophylaxis
PEP: Post-exposure prophylaxis
TasP: Treatment-as-prevention
U = U: Undetectable equals untransmittable
TGW: Transgender women
