# Supplementary material for: Why do men who have sex with men practice condomless sex? A systematic review and meta-synthesis
Source: BMC Infect Dis. 2022 Nov 14;22:850. doi: 10.1186/s12879-022-07843-z (PMC9661788; doi:10.1186/s12879-022-07843-z)
Supplement: Supplementary file 1 — Additional file 1. Original findings of the included studies (N = 39). [file 12879_2022_7843_MOESM1_ESM.doc]

**S1. Original findings of the included studies (N=39)**

| **Author and year** | **Original findings** |
| --- | --- |
| Li et al., 2010 | 1. “Rouyu”(a Chinese word, refers to desire of physical flesh), having direct genital contact 2. “Just for fun”, getting sexual satisfaction 3. Regarding as a primary sign of special trust and intimacy, posing as a guideline of sexual relationships 4. Finding true love 5. Gaining confidence and a self-image 6. Escaping from daily stressors 7. Feeling condom is a tool solely for disease prevention, 8. Being served as a metaphor of an inferior relationship 9. Preventing reduction of physical sensation and diminishment of psychological and sexual pleasure 10. Seeing condom as a proof of promiscuity 11. Seeing using condom as a sign of being a high-risk man 12. Determining a partner’s HIV status by reading physical and character traits 13. Seeing a man from rural area as “less polluted” than a city dweller 14. Seeing a good body condition within the context of clean and comfortable circumstance as an indicator of a safer sex partner 15. Promoting unsafe sex by “bad mood” or “negative emotions” |
| Moen et al., 2013 | 1. Considering “bare sex” superior to condom-protected sex 2. Subduing enjoyment, separating lovemaking bodies and persons, bringing bodies apart. 3. Reducing sensitivity, blocking direct body-to-body contact, decreasing pleasure, delaying ejaculation, and precluding the giving and receiving of semen by using condom 4. Effecting the potency and abilities as a lover, bringing sexual relationship to a halt by using condom 5. Enjoying 100% corporeal sensation in bare sex 6. Causing discomfort, pain, and erection difficulties by using condom 7. Not convincing penile-anal HIV transmissibility 8. Believing ridding postcoital semen and using lubricating substances can be protective without condoms 9. Conflicting with sexual enjoyment 10. Not being perceived as essential for HIV protection 11. Making unprotected sex decision out of dialogue or conscious deliberation 12. Reducing confidence, integrity, love or passion, sexual excitement and reliability. 13. Being receptive position in penile-anal sex 14. Experiencing condom breakage or slippage and other quality deficits |
| Peterson et al., 2003 | 1. Wanting to have any exchange of body fluids 2. Having heat of the moment 3. Having oral sex without a condom, 4. Having multiple partners 5. Using alcohol 6. Having sex at the spur of the moment thinking 7. Taking away from sexual pleasure 8. Lacking knowledge 9. Having huge cost of condoms 10. Feeling embarrassed to suggest using condom 11. Using less in committed relationships 12. Assuming partner is HIV free |
| Taggart et al., 2017 | 1. Not using condoms under desires for intimacy, trust, and commitment 2. Feeling connected to a partner let them to not use condoms |
| Campbell et al., 2013 | 1. Having unplanned episodes of UAI 2. Discussing the health of HIV-positive partners: viral load, CD4 count, and ART adherence were considered to factors of non-condom use 3. Ending condom use by HIV testing 4. Having unplanned episodes of UAI 5. Defaulting to condom use without discussion (“just understood”) |
| Mustanski et al., 2014 | 1. Knowing or trusting the sex partner 2. Having marriage/monogamy relationship 3. Lying on relationship duration 4. Feeling embarrassed, and being given a dirty look by cashier if you are so young 5. Costing too expensive 6. Lacking knowledge of using condoms 7. Feeling stupid to figure out what kind to get 8. Being tested for HIV or STIs proves you “clean” as a reason to not use condoms 9. Decreasing pleasure, 10. Dismissing the sex intimacy and making it more of a business exchange 11. Perceiving unavailability 12. Having fear of parents 13. Having potential discomfort 14. Having no pregnancy possibility in male-to-male sex |
| Ostergren et al., 2011 | 1. Feeing discomfort; 2. Preferring to bareback; 3. Having sexual difficult and loss of erection or latex allergy 4. Knowing HIV status/Believed or confirming HIV negative 5. Knowing HIV-positive status 6. Making coitus interruptus 7. Being first sexual experience 8. Having lower risk for insertive partner 9. Being In the heat of the moment; 10. Lacking availability of condoms 11. Experiencing condom broke or slipped off 12. Alcohol and/or drug use 13. Being effected by relationship and trust: a) In a monogamous relationship; b) In a relationship that may or may not be monogamous; c) Familiarity, trust and reciprocity 14. Being effected by interpersonal communication and dynamics： a) Resistance from partner; b) Mutual agreement; c) No discussion 15. Being effected by love, intimacy and desire |
| Neville et al., 2016 | 1. Viewing condoms use as HIV prevention 2. Being affected by HIV health promotion messages 3. Developing a judgemental attitudes toward condom use 4. Emerging a reactive culture that accepts condom non-use 5. Expressing a tension in decision-making around condom use/non-use: a) Condoms use means health issues; b) Seeing risk taking as an integral aspect of male sexuality 6. Identifying physical issues as a reason of condom non-use: a) Decreasing the inability to achieve and sustain an erection; b) Reducing sensitivity 7. Thinking HIV is curable and willing to take the risk 8. Being in long-term relationships, monogamous and closed partnership need not wear condoms 9. Viewing “top”(the insertive partner) is safer than “bottom”(the receptive partner) |
| Schnarrs et al., 2012 | 1. Releasing unavailability of condoms 2. Happening unplanned sex 3. Being caught in the moment 4. Using substance: alcohol and drugs 5. Lacking feeling, comfort and sensation 6. Being related to HIV, STDs and spreading disease: a) Viewing HIV as the most severe sexually health issue; b) Describing STDs as treatable and less severe; 7. Using condom less during oral sex 8. Having inability to become pregnant |
| Tadele, 2010 | 1. Having confusions and misunderstanding on HIV/AIDS 2. Believing HIV cannot be transmitted between two people with different blood types 3. Having defecated immediately, cleaning and washing as protective measure from HIV/AIDS 4. Thinking vaginal sex between a man and a women is risker than sex between two men 5. Perceiving semen contains no HIV virus, only blood does 6. Doing not engage in intercourse and not seeing the need of condoms 7. Making use of condoms is difficult in a no secure and private environment 8. Claiming mala-to-male does not transmit HIV/AIDS 9. Reducing the risks of getting HIV by small size of the gay population 10. Expressing a strong desire for “cool sperm” 11. Emphasizing the bare penis as “an important site of sexual sensation and erotic pleasure” 12. Using their own ways to prevent HIV infection 13. Being affected by religion and the crucifixion of Christ 14. Using substance: smoking, drinking and chewing khat Catha edulis |
| Li et al., 2016 | 1. Having inaccurate HIV knowledge: a) 2. Thinking oral sex was safe and did not require condom use 3. Thinking semen contains no virus; 4. Lacking perception of HIV prevalence: not knowing the extent of HIV prevalence in MSM in Changsha or China 5. Trusting the sex partner 6. Assessing partner health status by observation, touch, communication or HIV test 7. Having treatment optimism 8. Suffering hard life in reality 9. Perceiving HIV and homosexual stigma 10. Feeling discomfort 11. Lacking condom availability 12. Affecting trust and love by using condoms 13. Having difficulty on maintaining one partner for a long time 14. Using condom in oral intercourse is rare 15. Knowing condoms can only prevent HIV/STI 16. Lacking self-control 17. Lacking negotiating capacity |
| Beoughe et al., 2012 | 1. Drawing a line between the couples 2. Deciding having sex without condoms would be a tolerable risk area 3. Leading the discussion of condom use by HIV-negative partner 4. Choosing to forgo condoms is grounded in the couple’s level of acceptable risk, sexual preferences, and personal feelings 5. Having less risky for the top than the bottom in unprotected sex 6. Destroying good and natural sex by using condom 7. Seropositioning (i.e., the HIV-negative partner is insertive) 8. Monitoring the HIV-positive partner’s viral load 9. Withdrawing before ejaculation 10. Avoiding sex when sick or injured (e.g., cut in mouth) |
| Harawa et al., 2006 | 1. Lacking availability, particularly in homeless shelters and incarcerated settings 2. Distrusting the effectiveness of condoms for disease prevention 3. Having aesthetic concerns regarding condom use 4. Taking away from the feel of the pleasure of having the actual flesh and reducing the pleasure and senses by using condom 5. Having unprotected sex to avoid losing the partner and the payment 6. Using drugs and alcohol |
| Valente et al., 2019 | Barriers to condom use reported by MSW:   1. Having extra-payment 2. Being forced by violence 3. Perceiving low-risk clients 4. Being drunk or high 5. Engaging in group sex   Barriers to condom use reported by MCM:   1. Decreasing sexual sensation and pleasure 2. Being drunk or high 3. Engaging in group sex 4. Perceiving low-risk MSW 5. Having no condoms with them |
| Chakrapani et al., 2013 | 1. Thinking the use of condoms was stigmatized in front of children 2. Not always carrying condoms 3. Being shamed for carrying and using condoms for unmarried man 4. Being embarrassed to ask for condoms from the chemist shop 5. Developing an instant trust because they are from the “same circle” or “same class” 6. Being forced to have unprotect sex 7. Suffering sex abuse 8. Having intimacy, love, ‘long-term’ relationship and trust with their regular partners 9. Trusting sexual partner 10. Having familiarity and reliance on the self-reported monogamy 11. ‘Releasing the heat’ during sex 12. Release the semen into the anus is seen as necessary 13. Having skin-to-skin contact means having “real sex” 14. Perceiving good-looking partner have no any infections 15. Assessing partner by external appearance 16. Seeing Muslim partners as clean partner 17. Lacking knowledge of condom and being scared to use condoms 18. Being difficult to get or maintain erection when wearing condoms |
| Adam et al., 2010 | 1. Having erectile difficulties with condoms 2. Having risk "reduction" practices: withdrawing; or not ejaculating in a partner. 3. Assuming the top role in unprotected sex; 4. Being a top role 5. Having “heat of the moment” scenarios and the urgency of passion. 6. Meaning the opportunity to connect with a particularly desirable partner. 7. Using drugs and alcohol 8. Suffering personal turmoil and depression 9. Having stressful events and low self-esteem 10. Being affected by disclosure and intuiting safety 11. Using intuition or interpreting cues and signs in an attempt to discern the sero-status of a partner 12. Coining the term "negotiated safety": having unprotected sex after ascertaining sero-status and mutual agreement 13. Concerning romance, trust, and caring that "just happen" 14. Being affected by monogamy 15. Reading unprotected sexual practices as a sign of the special trust 16. Reading adopting protection as an accusation of infidelity 17. Seeing drop condom use as a sign of the serious relationship. 18. Being affected by the promise of intimacy 19. Being affected by romantic engagement 20. Recapturing freedom by barebacking 21. Having long-term unsatisfactory experience with condoms 22. Diminishing sexual satisfaction 23. Denying the discovery or rediscovery of eroticism by condoms 24. Being affected by micro- cultures of barebacking |
| Adam et al., 2000 | 1. Involving agreement of non-condom use (either tacit or verbalized) between two HIV-negative men. 2. Reading unsafe sex as a sign of the special trust in a couple 3. Being unconcerned about contracting HIV from closer partner 4. Occurring within an ongoing relationship or monogamy script 5. Viewing unsafe sex as a means of expressing or maintaining a feeling of intimacy or romance. 6. Having unintended unsafe sexual encounters 7. Lacking knowledge or access to information. 8. Determining a partner's HIV status by reading physical and character signs 9. Acting accidental unsafe sex due to condom breaking 10. Being associated with depression or a negative mood state 11. Using unsafe sex as an escape from everyday life 12. Using drugs and alcohol to heighten a sense of abandon 13. Submitting to "heat of the moment": enjoying sex and the pleasure and the excitement of the moment 14. Reading implicit signs of a partner's HIV status as a method of ascertaining safety 15. Intuiting a sense of trust and safety from partner's youth, connections with heterosexuality, and by adopting the insertive role in anal sex 16. Intuiting safety of partner by sexual history (such as amount of female partners), view heterosexual as unsafe |
| Adams et al., 2009 | 1. Using alcohol and other drug 2. Having low self-esteem 3. Offering emotional connectedness as a powerful factor 4. Feeling connected to the person by non-condom use 5. Seeking excitement: talking about anal sex without condoms as something that was exciting, risqué, exhilarating, and thrilling 6. Having psychological difficulties associated with using condoms: diminishing the pleasure and enjoy of sex 7. Spoiling the “heat of the moment” 8. Having physical difficulties associated with using condoms: using condoms is not cooler 9. Having physical difficulty of staying “hard” (maintaining an erection) 10. having strong skepticism toward health promotion messages encouraging condom use 11. Utilizing monogamy as a way to manage the risk of being exposed to HIV among gay couples 12. Achieving a level of trust and openness with somebody 13. Drawing on the concept of “negotiated safety”: establishing trust in relationships and making and establishing agreements 14. Being the insertive partner 15. Having an HIV test 16. “Looking healthy” |
| Balán et al., 2009 | 1. Thinking bareback sex as more intimate and emotionally satisfying than sex with condoms 2. Viewing bareback sex as an “entire connection with the person you are with, feeling every part of them, without interruption 3. Deriving greater pleasure from condomless sex owing to increased physical sensation 4. Making receptive anal sex more painful owing to drying out with condoms 5. Decreasing fear of dying of AIDS by available treatments. 6. Taking steps to reduce the risk of HIV infection: choosing partners of reportedly same serostatus 7. Using alcohol or drugs 8. Being affected by homophobia, machismo, and denial of homosexuality 9. Being affected by economic difficulties 10. Using alcohol and drug 11. Being affected by ethnic identification |
| Boulton et al., 2010 | 1. Expressing love and intimacy without condoms 2. Heightening expression of mutual commitment without condoms 3. Being affected by the power of sexual desires, particularly in the heat of an especially erotic moment 4. Using condoms reduce sexual satisfaction 5. Believing that neither they nor their partner were infected 6. Having negative HIV tests 7. Knowing partner’s sexual history in detail 8. Being in a long term relationship 9. Judging whether the partner was HIV-positive by his personal qualities, such as prestigious social characteristics 10. Looking to more specific situational factors as evidence that their partner was ‘not a risky person’ 11. Lacking knowledge: withdrawing before orgasm was safe 12. Viewing active as safe rather than passive intercourse 13. Using condom could be taken as implying that the interviewee thought that either he or his partner could be HIV positive. 14. Using condom could be taken as implying that either the interviewee or his partner had unprotected intercourse with someone else in a mutually monogamous relationship 15. Being against by stigma and social pressures 16. Being difficult to know how to ask partner to use a condom 17. Using alcohol or drugs 18. Having grief and emotional distress 19. Being forced by partner |
| Diguez et al., 1996 | 1. Diminishing pleasure 2. Having low risk perception 3. Having trust in and emotional connection with partner 4. Suffering unavailability/inconvenience of condoms 5. Lacking control (passion, excitement, impulsivity) 6. Using substance 7. Being Indifferent/ignorant about safer sex 8. Having communication problems/omission |
| Eisenberg et al., 2011 | 1. Creating a connection based on trust and intimacy by UAI (unprotected anal intercourse) 2. Marking a relationship had transitioned from a casual to a serious relationship via UAI 3. Engaging in UAI as a strategy to create a pleasurable connection 4. Describing UAI was a way to communicate feelings of trust with a partner 5. Reflecting commitment to a partner 6. Occurring miscommunication during discussion of condom use 7. Broking a proceeding sexual encounter 8. Keeping silence about condom use 9. Utilizing nonverbal health cues ( such as smell and taste) and visual markers of health (e.g., pigments or marks in their body) as a technique to assess the health of partners. 10. Losing pleasure when wearing a condom |
| Giano et al., 2019 | 1. Feeling discomfort 2. Lacking feeling 3. Having difficulty achieving/maintaining an erection 4. Inhibiting sexual pleasure 5. Trusting their partner 6. Being honest about their HIV and STI status and believing their risk of infection to be minimal 7. Having an exclusive sexual relationship 8. Viewing sexual monogamy as a buffer from the risks of HIV/STI acquisition within the relationship 9. Viewing the absence of condoms in sexual intercourse as enhancing relationship bonds 10. Not using condom during oral sex 11. Employing a pulling-out method before ejaculation to minimize fluid exchange 12. Using alcohol and/or other substances 13. Using “meth” (methamphetamine) during sexual activity 14. Knowing their partner’s HIV /STI status and serosorting behavior 15. Having HIV/STI test results 16. Knowing someone well enough 17. Getting tested every few months 18. Suffering physical and verbal abuse 19. Having a gap in sexual education among MSM 20. Having varying reasons related to the cultural aspect |
| Harawa et al., 2010 | 1. Diminishing either the pleasure or excitement of sex. 2. Taking the opportunity when it arises even without condom 3. Having committed relationships 4. Viewing sex without a condom as a sense of trust and bonding 5. Making decision of condom use by observing other inmates and listening to gossip 6. Being limited on the assumption of condom 7. Willing to infect others due to the resentment and bitterness they have about contracting HIV 8. Having feeling of ignorance, hopelessness, and bitterness |
| Harper et al., 2016 | 1. Decreasing pleasure with use of a condom 2. Focusing on the act of sex and the immediate gratification from that act 3. Having too big penises to have condoms 4. Lacking knowledge: condoms are only for pregnancy prevention |
| Hospers et al., 1994 | 1. Finding condoms too expensive or hard to acquire. 2. Decreasing sexual pleasure 3. Losing intimacy 4. Having difficulties of using condom with regular partner 5. Feeling less responsible for their sexual partners in anonymous situations and accepting certain risk 6. Being in love 7. Not wanting to disappoint their partner 8. Using alcohol 9. Naming their psychological well-being as an important reason for risk-taking behavior: worthlessness, or loneliness, or being unhappy 10. Expecting there would be a vaccine or treatment available |
| Hubach et al., 2014 | 1. Increasing pleasure expectations without condoms 2. Being hard to access condom 3. Using substance 4. Having no fears of unintended pregnancy 5. Having UAI with primary or established partners |
| Klassen et al., 2019 | 1. Inhibiting physical pleasure/ having desire for increased sexual pleasure / decreasing physical sensation 2. Having desire for eroticism and freedom 3. Using substance 4. Having perception of low risk 5. having treatment optimism and perceived efficacy interventions 6. Maintaining the mood or the “heat of the moment” 7. Discussing sexual safety and potential condom use disrupted or inhibited sexual interactions 8. Having imbalances in relationship power dynamics and ceding sexual decision-making to partners 9. Having peer pressure and the threat of withholding sex 10. Pursuing emotional intimacy, closeness and trust 11. Getting tested and agreeing to monogamy with a partner 12. Enjoying “barebacking” 13. Having seroadaptive strategies 14. Being affected by the availability and quality of HIV treatment 15. Seeking the effective means of prevention |
| Kong, 2008 | 1. Having incorrect AIDS knowledge and cultural myths 2. Having economic hardship (Financial reasons) 3. Having strong discrimination against homosexual orientation 4. Having over-trusting in sexual relationships |
| Malebranche, 2009 | 1. Having “Trust” on their partner 2. Viewing the decision to not utilize a condom as a much more intimate act 3. Being a “top” role in sex behavior 4. Coming down to “heat of the moment” sex 5. Suffering poor education 6. Being influenced by “low self-love” |
| Musinguzi et al., 2015 | 1. Concerning about the quality of condoms and lack of lubricants 2. Feeling discomfort and pain 3. Viewing accessing condoms as a barrier to condom use 4. Viewing affordability as a barrier to condom use 5. Lacking knowledge about condoms or how to use condoms 6. Suffering condom-related fears and homophobia: putting the homosexual stigma on condom 7. Believing that sex with a fellow man is safe 8. Trusting the partner 9. Having weak safer sexual negation skills: using condoms means no longer trust 10. Having unplanned sexual intercourse 11. Judging the partners whether infected with HIV according to their appearance 12. Getting a higher pay for unprotected sex among the male sex workers 13. Using alcohol 14. Desiring sexual pleasure, exploration and being in a hurry |
| Middelthon, 2001 | 1. Reading responsibility and irresponsibility, respect and disrespect, and humiliation into the use and nonuse of condoms 2. Interfering with feelings of intimacy 3. Interfering with the ability to give, receive, or feel semen 4. Viewing nonuse of condoms among steady partners as a sign of trust 5. Viewing nonuse of condoms as a sign of trust and "difference"(this particular relationship is different from other relationships) |
| Siegler et al., 2014 | 1. Mixing alcohol and sex 2. Experiencing condom failure 3. Decreasing pleasure or negatively influence performance with condoms 4. Ceasing condom use in trusted relationships |
| Starks et al., 2017 | 1. Thinking CAI as a sign of trust and intimacy 2. Preventing the intimacy brought about through fluid exchange by using condom 3. Getting “caught in the heat of the moment” 4. Ruining the mood and passion by using condom 5. Ceasing condom use only after HIV tests that confirm the couple’s status as concordantly negative 6. Practicing monogamy |
| Zhang et al., 2018 | 1. Lacking knowledge of condom use: being unaware of the necessity for condom use, lacking understanding of the purposes for condom use, lacking awareness of full condom use 2. Lacking awareness of risk of non-insertive sex and oral sex 3. Using alcohol and substance 4. Regular sexual partner: trusting each other 5. Impairing sexual function 6. Believing students have a low risk of infection, trusting older partners, 7. Being induced or deceived to have unprotected sex by older partners 8. Being asked to have unprotected sex by “1” |
| Wang et al., 2005 | 1. Lacking awareness of full condom use 2. Lacking condoms 3. Having difficulty on affording condoms 4. Affecting the relationship and trust of sexual partners 5. Believing sexual partner is health 6. Thinking insertive role will not be infected 7. Thinking anorectal irrigation can prevent infection 8. Feeling uncomfortable with condoms 9. Decreasing sexual pleasure 10. Worrying police officers punished condoms as evidence of sex trade |
| Zou, 2008 | 1. Not preparing condoms by both of them 2. Having difficulty on acquire condoms 3. Feeling shame to buy condoms 4. Feeling uncomfortable with condom 5. Thinking the material of condom is bad for health 6. Having fluke psychology 7. Lacking knowledge and awareness of safe sex behaviors 8. Having sex with familiar sexual partner 9. Viewing sex without condoms as a trust of sexual partner 10. Judging whether using condom or not by characteristics of sexual partner 11. Having no enough condoms 12. Being affected by ethnic culture 13. Being affected by economic factors 14. Using alcohol and substance |
| Ofreneo et al., 2020 | 1. Accepting condomless sex with person who is assumed to be educated, careful and responsible 2. Accepting condomless sex with personwho has good appearance and language 3. Accepting condomless sex with person who is good-looking, clean and healthy 4. Accepting condomless sex with straight or cisgender males 5. Accepting condomless sex with regular sex partner or exclusive partner 6. Considering condom is necessary during sex with person who is uneducated, irresponsible and careless person 7. Considering condom is necessary during sex with person who is not good-looking and unhealthy 8. Considering condom is necessary during sex with non-straight male partners, particularly gay and bisexual partners, unfamiliar sex partners, and people with multiple sexual relationships |
| Rwstar et al., 2019 | 1. Having condom-related stigma among friends (social barrier) 2. Lacking comprehensive sex education and HIV curriculum (structural barriers) 3. Having condom-related stigma in school (social barrier) 4. Lacking outreach capacity to disseminate information about free cost, out of stock, HIV testing requirements 5. Having condom-related stigma in healthcare facilites (social barrier) 6. Affecting by inconspicuous arrangement and placement of condoms, distance to stores, and relative cost (structural barriers) 7. Having condom-related stigma in convenience stores (social barrier) 8. Affecting by unsupportive stance and prohibiting condom distribution activities (barriers) 9. Having condom-related stigma in church (barrier) |
